# A Few Items of Importance

**Published:** 1888-11

**Authors:** E. Parmley Brown

**Affiliations:** Flushing.


ARTICLE IV.

### A FEW ITEMS OF IMPORTANCE.

BY DR. E. PARMLEY BROWN, FLUSHING.

[From the Committee on Dental Practice in N. Y. Society.—E. Parmley Brown, C. F. W. Bodecker, W. C. Barrett.]

THE PERRY SEPARATOR.—The Perry separator has come, and come to stay, doing much good where properly filling its mission ; but being also capable of doing much harm and becoming in the hands of the reckless an instrument of torture when used where not indicated. Many days of soreness to the parts about the teeth on which it is used may follow, but skillful work by the operator may in most cases enable him to dispense with extensive separating. The highest degree of skill may be displayed by the best restoration with the least degree of separation, the desired result of close knuckling belng equally well attained. The separators are specially valuable where time cannot be had for securing space with cotton ; but your chairman has adopted the universal practice of late years, of packing cotton between the teeth for a slight separation which is thereby obtained with the least discomfort of any system that he has ever seen, and at the same time fulfilling the double mission of producing space and driving the gum away from the locality of the work by the expansion of the cotton fibres—a few drops of Calvert's No. 1 carbolic acid and oil of cloves, in equal parts, being applied to the cotton when so used. This medicament assists materialy in obtunding and allaying irritation of the parts. In very close contact, where it is desirable to use the cotton, it may be thoroughly placed by being driven between the teeth with a steel wedge and a hand mallet.

THE KNAPP BLOW-PIPE.

The late birth of the powerful Knapp blow-pipe, where by nitrous oxide and illuminating gas are united in marriage, producing the hottest thing yet known to the dental world, and facilitating the use of porcelain and the metals in crown and bridge-work, is another advanced step in the march of our profession. The blow-pipe has been perfected by the manufacturers; and the small furnace for baking teeth, in connection with it, we are promised immediately. Though the blow-pipe may be too powerful for ordinary soldering work, yet for supplying heat for the new furnace for porcelain work it promises to prove a great blessing.

THE E. PARMLEY BROWN PORCELAIN BRIDGEWORK.

After two years' constant use of the E. Parmley Brown porcelain bridgework, your Chairman wishes to say unhesitatingly that he believes that he has builded even better than he knew. In mouths where the gold bridgework could not be tolerated an account of a diseased condition of the gums, produced by contact with the metal, the porcelain in contact with the gums is tolerated without the slightest irritation; but exactly why this is so, the inventor is not as yet prepared to state. Recent examinations of several cases inserted over two years ago encourage the inventor more than his most sanguine expectations promised. The anchorage of short bridges into strong, firm teeth, by embedding the bar running through the porcelain teeth into solid gold fillings, is strong and satisfactory beyond the belief and comprehension of those who have never done the work nor seen it done thoroughly; and further, for natural appearance inside and outside of the arch, there is nothing that approaches it. This system of bridgework may be used in all its varied ways without infringement on any other patents, and will be withheld from the unprofessional advertisers, being licensed, at a moderate fee, to those who practice according to the Code of Ethics.

### COCOAINE.

Your committee seem somewhat divided in its opinion of the recentiy introduced cocoaine. Dr. Bodecker pronounces it of no value in the excavation of teeth, but of great service for use on the soft tissues, the removal of deep-seated calculary deposits and necrosed bone. Your chairman found it of no value in the excavation of sensitive teeth till used in connection with carbolic acid and oil of cloves, the cocaine being carefully kept from the margins of the cavity, as the hydrochloric acid in the solution has great affinity for the lime salts in the tooth-substance, enamel, or dentine; and he has found by experiments on some difficult cases of excavation where the sound tooth-substance had to be removed for the anchorage of bridgework, that the application of these obtundants —cocaine, carbolic acid, and oil of cloves—not only relieves the temporary aching of the tooth (where the rubber dam is applied), but that they soften the tooth-substance, making excavation painless to the patient and easy for the operator till the zone of affected tooth-material is removed. Your Chairman has found the cocaine valuable in two other operations of great importance to the dentist: In connection with arsenic where a diseased pulp is to be destroyed and removed in a few days, none of the usual tormenting pains attending or following the application; and also where it is desirable to remove a living pulp immediately—a few applications of the cocaine making the operation painless in a few moments.

### THE PERRY-WEBER DENTAL ENGINE.

The Perry-Weber dental engine seems to be a great and valuable addition to dental machinery.

### IMPLANTATION.

Replantation and transplantation seem to have come to life again, and implantation to be a child born to them. Replantation, it is well known, has been successful in but a small percentage of cases in the past; and transplantation successful in but a very small percentage. In implantation Dr. Bodecker says he has little faith as yet. He does not believe a new socket is formed; and he is trying to obtain a section of a socket in which a tooth has been implanted, to examine under the microscope, in which he has not succeeded, owing to obvious difficulties. Dr. Bodecker's opinion in regard to implantation is that the temporary union between the tooth and the walls of the newly made socket is merely a mechanical cementation, in which opinion your chairman coincides; and he may astonish you by stating that he performed the operation of implantation seventeen vears ago when a superior central incisor was implanted in the place of a split " nubbin " of a superior lateral just extracted, because the space was much too wide for a lateral, and the socket had to be considerably enlarged to receive the central. A new socket was thereby practically made. Success was promised at first, but in a year or more the tooth came out suddenly and without any warning, though the root and the surrounding parts were in a healthy condition.—*Items of Interest.*

